# Quantitative Evaluation of Pulsed Thermography, Lock-in Thermography and Vibrothermography on Foreign Object Defect (FOD) in CFRP

**DOI:** 10.3390/s16050743

**Published:** 2016-05-21

**Authors:** Bin Liu, Hai Zhang, Henrique Fernandes, Xavier Maldague

**Affiliations:** 1 School of Information Science and Engineering, Shenyang University of Technology, 111 Shenliao West Road, Shenyang 110870, China; syuotwenwu@sina.com; 2 Department of Electrical and Computer Engineering, Computer Vision and Systems Laboratory, Laval University, 1065 av. de la Médecine, Quebec City, QC G1V 0A6, Canada; henrique-coelho.fernandes.1@ulaval.ca (H.F.); xavier.maldague@gel.ulaval.ca (X.M.); 3 Department of Mechanical Engineering, Federal University of Uberlandia, 2121 Av. Joao Naves de Avila, Uberlandia 38400-902, Brazil

**Keywords:** CFRP, FOD, lock-in thermography, pulsed thermography, vibrothermography

## Abstract

In this article, optical excitation thermographic techniques, including pulsed thermography and lock-in thermography, were used to detect foreign object defect (FOD) and delamination in CFRP. Then, vibrothermography as an ultrasonic excitation technique was used to detect these defects for the comparative purposes. Different image processing methods, including cold image subtraction (CIS), principal component thermography (PCT), thermographic signal reconstruction (TSR) and Fourier transform (FT), were performed. Finally, a comparison of optical excitation thermography and vibrothermography was conducted, and a thermographic probability of detection was given.

## 1. Introduction

Carbon fiber-reinforced polymer composite (CFRP) is being increasingly utilized in aircraft, vehicles, ships, and sports equipment, *etc.* The reason for its wide usage is the significant weight reduction at the same strength [1]. However, the flaws that occur during manufacturing seriously threaten public security. Therefore, it is important to develop inspection techniques to assess the material. Destructive techniques, such as microscopic inspection, are effective, but will destroy the material, so that it is not practical to conduct in-line detection [2]. Non-destructive testing (NDT) techniques are a better replacement compared to destructive methods.

Compared to ultrasonic c-scan [2], magnetic flux leakage [3] and X-ray computed tomography [4], infrared thermography (IRT) is increasingly used as a NDT technique due to its fast inspection rate, being contactless, having high spatial resolution, improved acquisition rate and the development of infrared image processing. Optical excitation thermography is well known as a traditional infrared thermography technique. However, it is difficult to detect flaws in thick CFRP. Recently, vibrothermography as an ultrasonic excitation thermography is becoming increasing popular in the inspection of CFRP; for example, it was used to detect cracks by the authors and provided positive results [5]. To better extend the usage of the technique, it is important to increase its applications to other types of flaws.

Compared to pulsed thermography and lock-in thermography, which were already comprehensively studied, the detection capacity of vibrothermography can be further explored. In particular, the comparative study on FOD of vibrothermography stimulated at different positions was poorly documented in the open literatures. In this article, optical excitation thermography techniques including pulsed thermography and lock-in thermography, were used to detect foreign object defect (FOD) and delamination in a CFRP specimen. Then, vibrothermography as an ultrasonic excitation thermography technique stimulated at different positions was used to detect these defects for comparative purposes. Different image processing methods, including phase image Fourier transform (FT), cold image subtraction (CIS), principal component thermography (PCT) and thermographic signal reconstruction (TSR), were performed. Finally, a comparison of optical excitation thermography and vibrothermography was conducted, and a thermographic probability of defection (thermoPoD) was given.

## 2. Specimen Description

**Figure 1:**
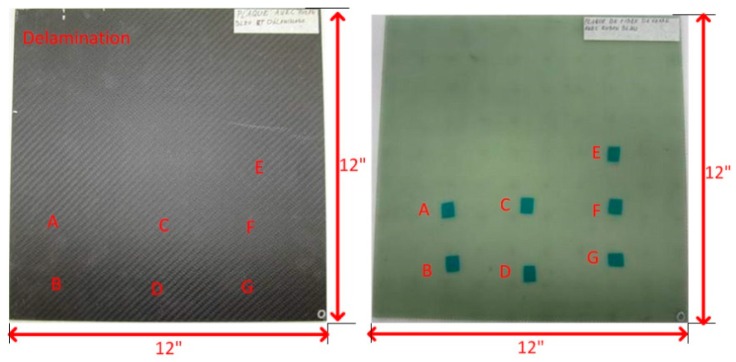
(**a**) The experimental CFRP specimen; (**b**) The corresponding CFRP specimen.

The experimental 12${}^{\prime \prime }$ × 12${}^{\prime \prime }$ CFRP specimen is shown in Figure 1a. Figure 1b shows the corresponding GFRP model, where the foreign object defect (FOD) can be seen clearly. For CFRP, twill 2 × 26 K carbon fiber was used. For GFRP, plain weave 7500 glass 9.60 oz/yd2 was used. Both the CFRP and GFRP specimens were made by infusion with epoxy resin. A post cure at 85 ${}^{\circ }$C was conducted to ensure the complete crosslinking. The stacking sequence is 0 ${}^{\circ }$C for everting.

The specimens contain eight plies. For the CFRP specimen, the thickness of each ply is about 0.0166${}^{\prime \prime }$, and for the GFRP specimen, it is about 0.0125${}^{\prime \prime }$. A Teflon sheet was used for the delamination. The thickness is 0.0025${}^{\prime \prime }$. After the infusion, the sheet was taken off to imitate a delamination. For the FODs, flash breaker tapes made of polyester with silicone surface coat were used. The flash breaker tapes were spaced against one of the layers of prepreg during the layup.

The FODs are between each ply. The eighth ply is on the surface of the mould, and the fist ply is the rough surface. The FODs are marked as follows:

- Defect A: between Ply 1 and 2
- Defect B: between Ply 2 and 3
- Defect C: between Ply 3 and 4
- Defect D: between Ply 4 and 5
- Defect E: between Ply 5 and 6
- Defect F: between Ply 6 and 7
- Defect G: between Ply 7 and 8

## 3. Infrared Image Processing

### 3.1. Cold Image Subtraction

Cold image subtraction (CIS) is intended to decrease the impact of fixed artefacts in thermographic images. These may include the reflections from the circumstances, such as remaining heating from the lamps, the reflection from the camera, which appears in the progress of the acquisition, *etc.* Because these artefacts do not change, including before heating when the image is cold in the progress of the acquisition, the image or the average of a few images can be subtracted before heating, which allows their effect to be decreased [4].

### 3.2. Principal Component Thermography

Principal component thermography (PCT), originally proposed by Rajic [6] in 2002, extracts the image features and reduces undesirable signals. It relies on singular value decomposition (SVD), which is a tool to extract spatial and temporal data from a matrix in a compact manner by projecting original data onto a system of orthogonal components, known as empirical orthogonal functions (EOF). The first EOF will provide the most important characteristic variability of the data; the second EOF will provide the second most important variability, *etc.* Original data can often be adequately represented with only a few EOFs. Usually, an infrared sequence of 1000 images can be replaced by 10 or less EOFs [7].

### 3.3. Thermographic Signal Reconstruction

Thermographic signal reconstruction (TSR) [8] gives a key degree of data compression because only polynomial coefficients are reserved. It is also convenient to produce derivative images without additional noise generations. Conducting first and second time derivatives of TSR gives an obvious improvement in signal to noise performance and provides a good sensitivity to defects with a smaller size and a deeper depth [9].

## 4. Optical Excitation Thermography

In optical excitation thermography, light is transformed into heat, and energy is transferred to the specimen surface. Usually, an infrared camera is used to record the specimen surface temperature profile. The subsurface discontinuities may change the heat diffusion, which therefore affects the cooling procedure of the nearby zone on the surface [10]. The representative optical excitation methods include pulsed thermography and lock-in thermography.

### 4.1. Pulsed Thermography

Pulsed thermography is a classical optical excitation thermography technique. In pulsed thermography, high-energy lamps are often used to produce a uniform heating source on the specimen surface. The heat transmits through the inspected specimen to the subsurface anomalies, such as defects or damages, and then returns to the specimen surface. When the pulsed heat flux is delivered to the specimen surface, an out-of-plane heat flow is produced in the specimen. A uniform temperature rise will be recorded if there are no defects in the specimen. If there are defects such as voids or delamination, a localized high-temperature zone will be observed above the defect due to the insulation effect. The shape of the high-temperature zone represents the defect shape. The location, shape and size of the defect can be estimated from the temperature distribution on the sample surface [11,12].

### 4.2. Lock-In Thermography

Lock-in thermography, derived from photo thermal radiometry, is also well known as a modulated technique. In lock-in thermography, the absorption of modulated optical heating leads to a temperature modulation, which transmits through the specimen as a thermal wave. When the thermal wave is reflected by the defect boundary, the superposition to the original thermal wave will lead to the transformation of the response signal amplitude and phase on the surface. These signals are simultaneously recorded by the IR camera [13].

Fourier transform is an interesting image processing method for lock-in thermography, because it allows phase and amplitude data to be retrieved from the temperature-time history of each pixel. From Fourier’s Law’s one-dimensional solution for a periodic thermal wave transmission through a semi-infinite homogeneous material, the thermal wave diffusion length is given by [14]:

(1) $$\mu =\sqrt{\frac{2\alpha }{\omega }}=\sqrt{\frac{\alpha }{\pi f}}$$

where $\alpha =\kappa /\rho c_{p}$ is the material diffusivity, *κ* is the thermal conductivity, *ρ* is the density, $c_{p}$ is the specific heat (at constant pressure) and ω=2πf is the modulation frequency.

The detection depth *z* is given by the thermal diffusion length equation [15]:

(2) $$z=C_{1}\mu$$

where $C_{1}$ is a correlation constant. The reported values of $C_{1}$ range from 1.5 to 2 [15,16].

It is obvious that the detection depth depends on the lock-in frequency: the lower the lock-in frequency, the deeper the detection depth. Usually inspections begin from a high lock-in frequency, which depends on the material thermal diffusivity. Then, the lock-in frequency decreases gradually to access the deeper depth until an appropriate value is reached.

In this article, different lock-in frequencies from 3 Hz to 0.02 Hz were used to reach different depths. To estimate the detection depths, $\alpha =4.2\times 10^{-7}$ m${}^{2}$/s, which was measured by Ibarra-Castanedo [17], was adopted for CFRP. $C_{1}=1.8$, which is most frequently presented, was adopted. Based on these values, Table 1 shows the estimated detection depths, which are approximate.

Phase images are often more preferred for analysis than aptitude images due to the tolerance to the non-uniform heating, emissivity variations and circumstance reflections [11]. Usually, they can also provide deeper detection depth than amplitude images. In particular, analysing phase data obtained from fourier transform to obtain improved inspection results in pulsed thermography is also known as pulse phase thermography [18], which can even be thought of as being a combination of pulsed thermography and lock-in thermography. In a similar method as for lock-in thermography, phase data are usually analyzed, due to its tolerance to the non-uniform heating and circumstance reflections. In practice, usually a fast Fourier transform allows the signal to be processed more effectively [10,19].

The method usually used to retrieve phase and amplitude is a four-point methodology for the sinusoidal stimulation phase, which is fast, but works only for sinusoidal stimulation [20,21]. The four-point methodology is usually affected by noise, but the signal can be de-noised partly by averaging a few points. However, this de-noising method sharply slows down the calculations. Fourier transform can also be used to extract amplitude and phase images from lock-in data. The Fourier transform can be used for any waveform and is also able to de-noising signals [10]. Therefore, Fourier transform was performed to retrieve phase images from the raw images in this article.

### 4.3. Experimental Setup

**Figure 2:**
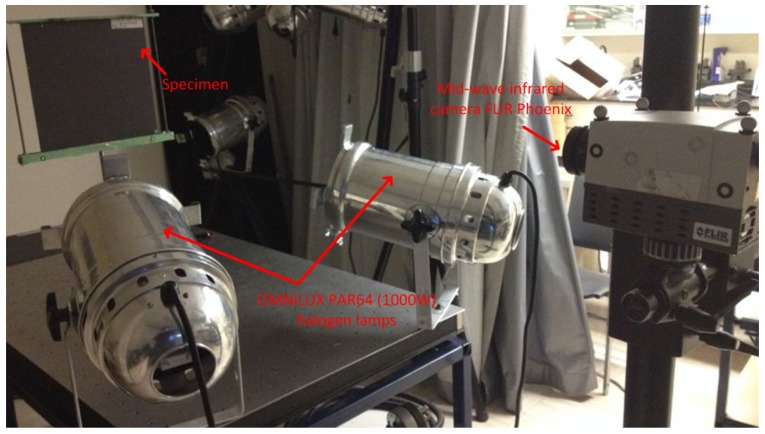
The optical excitation thermography experimental set-up.

Figure 2 shows the experimental set-up for optical excitation thermography. In the setup, two ’OMNLUX PAR64’ (1000 W) halogen lamps were used to generate sinusoidal or pulsed thermal waves. A mid-wave infrared camera ’FLIR Phoenix’ was used to record the temperature profile. Table 2 shows the important technical specifications of the camera. The lamps and the infrared camera were located on the same side with respect to the specimen front surface.

### 4.4. Analysis of the Results

**Figure 3:**
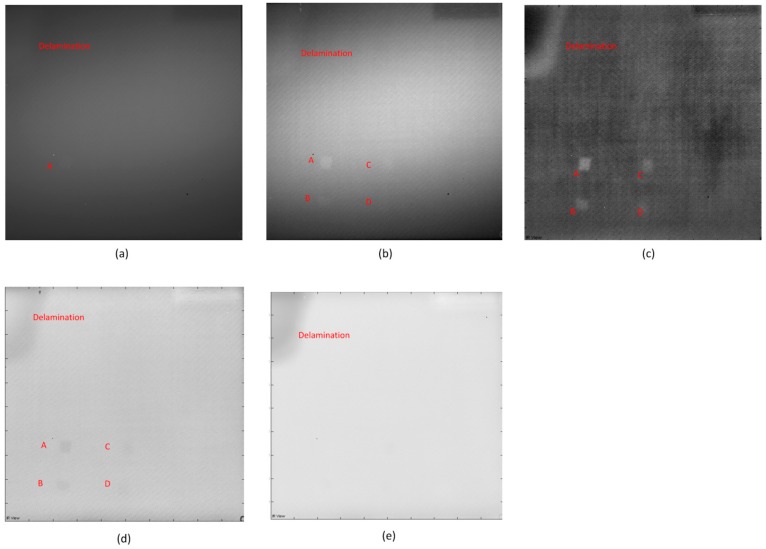
The pulsed thermography results: (**a**) Raw image; (**b**) After CIS; (**c**) PCT: EOF 5; (**d**) TSR: 1st derivative; (**e**) TSR: second derivative.

Figure 3 shows the pulsed thermography results. The experimental heating time (pulse length) is 30 s. Figure 3a shows the raw image, where only the blurry Defect A can be detected. In Figure 3b, more defects can be inspected after CIS. Figure 3c shows the image acquired from the fifth image (EOF 5) after PCT. In Figure 3c, these defects inspected in Figure 3b are clearer, and the position of the defect E is darker in color than other regions. This may indicate the probable existence of Defect E. Figure 3d,e shows the images after TSR. It is obvious that the 1st derivative result is clearer than the second derivative result, but PCT can provide the most inspection details and the best definition.

The authors increased the heating times to 120 s for pulsed thermography experiments. However, the longer pulse cannot provide better inspection results. According to the comparison of the images acquired from different image processing methods, PCT is the best solution for this type of defect using pulsed thermography.

**Figure 4:**
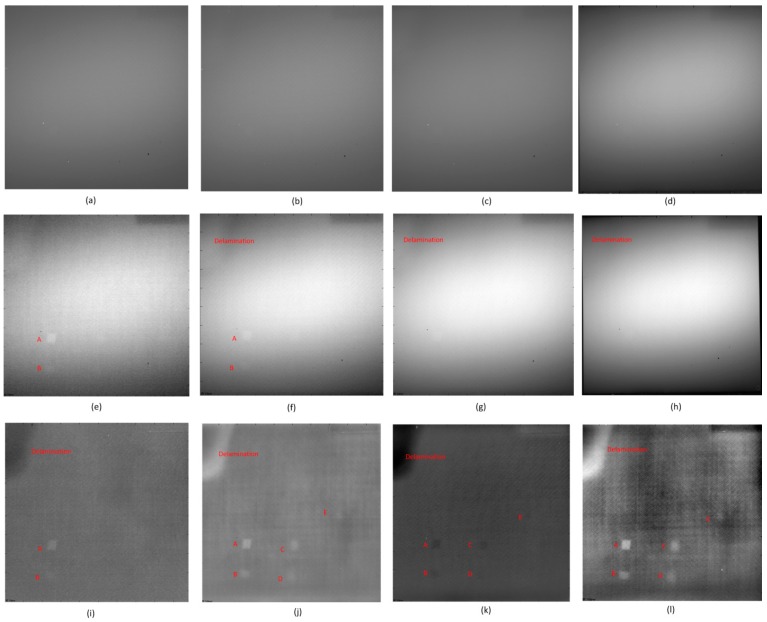
The lock-in thermography results, (**a**) Raw image: 3 Hz; (**b**) Raw image: 1 Hz; (**c**) Raw image: 0.4 Hz; (**d**) Raw image: 0.05 Hz; (**e**) CIS: 3 Hz; (**f**) CIS: 1 Hz; (**g**) CIS: 0.4 Hz; (**h**) CIS: 0.05 Hz; (**i**) PCT: 3 Hz, EOF 4; (**j**) PCT: 1 Hz, EOF 5; (**k**) PCT: 0.4 Hz, EOF 4; (**l**) PCT: 0.05 Hz, EOF 7.

Figure 4 shows the lock-in thermography results. It is obvious that the images after PCT are the clearest, shown in Figure 4i–l. The images after CIS are clearer than the raw images. However, neither the raw images nor the CIS images can provide better inspection results. Similar to the pulsed thermography results, PCT can provide the most inspection details and the best definition.

In particular, along with the lock-in frequencies decrease, less defects can be inspected in the images after CIS in Figure 4e–h. This phenomenon indicates that CIS is not a good solution to lock-in thermography, which can also be extracted from the corresponding theory of this image processing method. One the contrary, this phenomenon does not occur with PCT. Therefore, PCT should primarily be used to process the lock-in thermography results in practice, since it is fast and effective.

**Figure 5:**
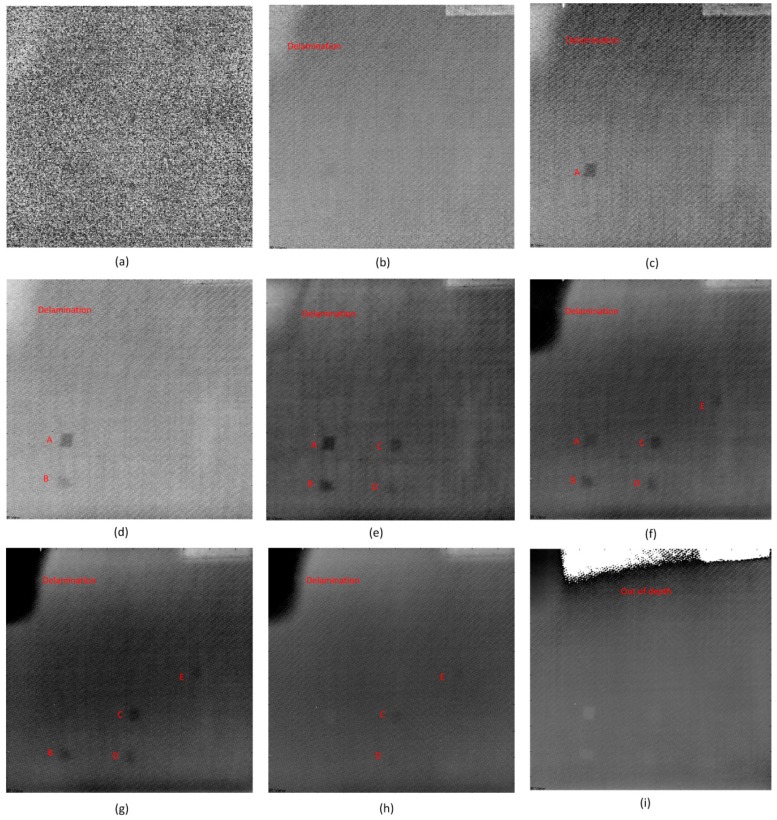
The phase images after Fourier transform: (**a**) Frequency: 3 Hz; (**b**) Frequency: 1 Hz; (**c**) Frequency: 0.6 Hz; (**d**) Frequency: 0.2 Hz; (**e**) Frequency: 0.1 Hz; (**f**) 0.05 Hz; (**g**) 0.04 Hz; (**h**) 0.03 Hz; (**i**) 0.02 Hz.

Figure 5 shows the phase images after Fourier transform with different frequencies, which are more tolerant to non-uniform heating. It is obvious that the non-uniform heating effects and noise (high frequency component) are reduced significantly in phase images. This image processing method can allow an estimation of the approximate detection depth.

In particular, when the lock-in frequency is 3 Hz in Figure 5a, the algorithm fails because the frequency is too high. When the frequency is 1 Hz in Figure 5b, Defect A is detected, but not clearly. This depth may be considered as the top surface of Defect A. When the frequency is 0.6 Hz in Figure 5c, Defect A is clearly detected. When the frequency is 0.2 Hz in Figure 5d, Defect B is also detected, but not clearly. This depth may be considered as the top surface of Defect B. In Figure 5e, Defect B is more clearly visible than in Figure 5d, and Defects C and D are also detected, but not clearly. In Figure 5f, Defects C and D are more clearly visible, but Defects A and B are not clear. Defect E is also detected in Figure 5f, but not clearly. This depth may be considered as the bottom surface of Defect A. When the frequency is 0.04 Hz in Figure 5g, Defect A disappears, and Defect E is still not clear. This depth may be considered as the bottom surface of Defect B. When the frequency is 0.03 Hz in Figure 5h, Defect A and B both disappear, and Defects C, D and E are all not clear. When the frequency is 0.02 Hz in Figure 5i, the algorithm fails because the estimated detection depth is beyond the actual specimen thickness.

Through Figure 5, the maximum detection depth of lock-in thermography is up to the depth of Defect E. When the frequency is lower, the deviation is greater. This technique can offer results at estimated detection depths using different lock-in frequencies, but only works well on the defects that are not too far from the surface.

Generally, both lock-in thermography and pulsed thermography are effective to detecting this type of defect. In particular, lock-in thermography can provide more inspection details than pulsed thermography after appropriate image processing. The fact that the Defect E is clearly inspected in Figure 4 and Figure 5 can strongly demonstrate this point. This defect cannot be inspected in Figure 3, which is acquired using pulsed thermography. However, neither lock-in thermography nor pulsed thermography can detect the Defects F and G, which are deeper than the other defects. Compared to traditional optical excitation thermography, ultrasonic excitation thermography might provide more detection results, which was also performed on this specimen by the authors.

## 5. Vibrothermography

Vibrothermography, also known as ultrasonic thermography, uses mechanical waves to directly stimulate internal defects without heating the surface as in traditional optical excitation thermography. In ultrasonic c-scan, a transducer is located in contact with the specimen through a coupling media, such as water. The ultrasonic waves travel through the specimen and are transmitted back to the specimen surface. To receive the reflective signals, usually, there are two methods. One solution is that the same transducer or another transducer receives the reflected signals on the same side, which is called the pulsed-echo technique. The other solution is that the signals are received by another transducer on the opposite side, which is called the transmission technique. The principle of defect detection is based on the differences in specific acoustic impedances between materials. In vibrothermography, the mechanism is totally different. Ultrasonic waves travel through a specimen as in ultrasonic c-scan. However, an internal defect results in a complex combination of absorption, scattering, beam spreading and dispersion of the waves. The primary presence of the waves is in the form of heat. Then, heat travels by conduction in all directions. Usually, the IR camera faces one of the surfaces of the specimen to acquire the defect information [12].

### 5.1. Experimental Setup

**Figure 6:**
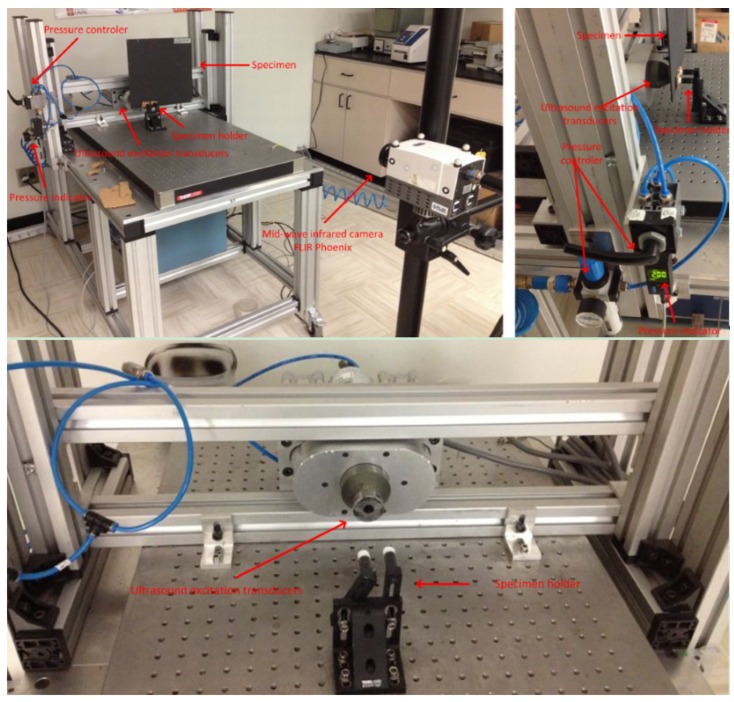
The vibrothermography experimental setup.

Figure 6 shows the experimental setup for vibrothermography. The IR camera ’FLIR Phoenix’ with the technical specifications in Table 2 was used again to record the temperature profile. In the set-up, the ultrasound excitation transducers were pressed against the specimen, and a burst of ultrasound waves was delivered to the specimen. Table 3 shows the important technical specifications of the ultrasound excitation setup.

In this article, the pulsed ultrasonic excitation was used, and the excitation time is 10 s.

### 5.2. Result Analysis

**Figure 7:**
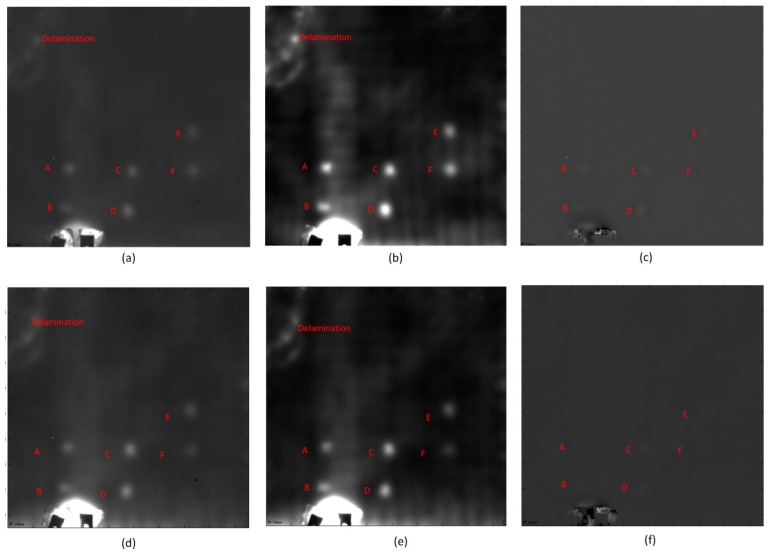
The vibrothermography results: (**a**) 50% power: raw image; (**b**) 50% power: CIS; (**c**) 50% power: PCT; (**d**) 80% power: raw image; (**e**) 80% power: CIS; (**f**) 80% power: PCT.

**Figure 8:**
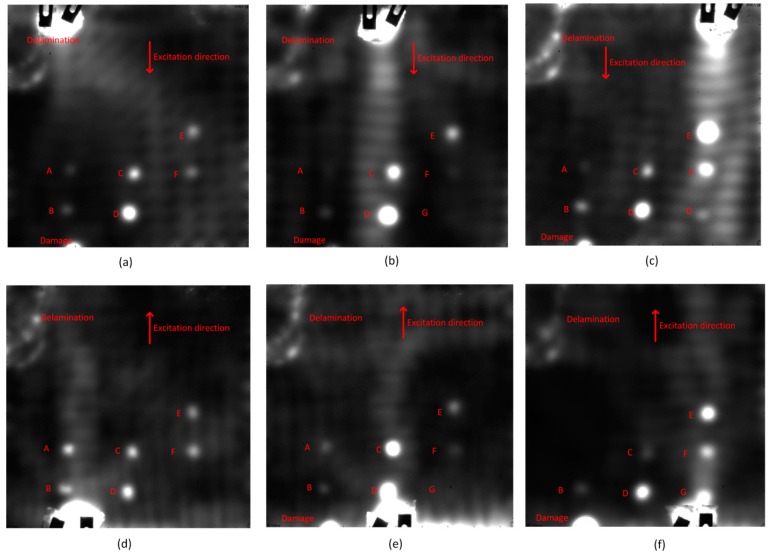
The complete vibrothermography results after CIS: (**a**) Left-top; (**b**) Middle-top; (**c**) Right-top; (**d**) Left-bottom; (**e**) Middle-bottom; (**f**) Right-bottom.

Figure 7 shows the vibrothermography results when the ultrasound excitation position is located at the left-bottom of the specimen. Contrary to the optical thermography results, the images after CIS are clearer. The images after PCT are blurry. When the ultrasonic excitation power is 50% of the maximum ultrasound excitation power, the defects A, B, C, D, E and F can be detected. A probable reason why Defect G cannot be inspected is that the ultrasound excitation power is low. Another probable cause is that the ultrasound excitation position is far from this defect. To find the cause, the authors increased the ultrasound excitation power to 80%. However, defect G still cannot be detected. The results are nearly the same as those with the 50% power. It is obvious that the increase of the ultrasound excitation power cannot increase the probability of detection, but may damage the specimen on the ultrasound excitation position shown in Figure 8. Therefore, the authors turned to the other probable solution.

Figure 8 shows the complete vibrothermography results with 50% of maximum power after CIS. The ultrasound excitation positions are respectively left-top, middle-top, right-top, left-bottom, middle-bottom and right-bottom. The delamination can be detected wherever the ultrasound excitation position is. Some interesting heat diffusion routes can also be inspected.

In Figure 8, when the ultrasound excitation positions are at the left-top and left-bottom, Defects A, B, C, D, E and F are detected, shown in Figure 8a,d. The Defects A and B in Figure 8d are more clearly detected than in Figure 8a. The probable reason is that the ultrasound excitation position is closer. However, an interesting phenomenon is that Defect D is more clearly detected in Figure 8a than in Figure 8d, which is closer to the ultrasound excitation position. The other interesting phenomenon is that Defects C and D in Figure 8a are more clearly detected than Defects A and B, which are closer to and in a line with the ultrasound excitation position.

When the ultrasound excitation positions are at the middle-top and middle-bottom, respectively shown in Figure 8b,e, Defects A, B, C, D, E, F and G are detected. Defects C and D are more clearly detected than the other defects. The probable reason is that defects C and D are closer to and in a line with the ultrasound excitation positions. Defect G is detected, but not clearly. Defects A, B, C, F and G are more clearly detected in Figure 8e than in Figure 8b. The probable cause is that they are closer to the ultrasound excitation position. However, an interesting phenomenon is that Defects D and E are more clearly detected in Figure 8b than in Figure 8e. This phenomenon also occurs when the ultrasound excitation positions are at the left-top and left-bottom, shown in Figure 8a,d. The other interesting phenomenon is that Defect F is not clear as in Figure 8a,d, although it is closer to and in a line with the ultrasound excitation positions.

When the ultrasound excitation position is at the right-top shown in Figure 8c, Defects A, B, C, D, E, F and G are detected. When the ultrasound excitation position is at the right-bottom shown in Figure 8f, Defect A cannot be detected. Defect G is more clearly detected in Figure 8f than in Figure 8c. The possible cause is that it is closer to the ultrasound excitation position. An interesting phenomenon is that Defects B, C, D, E and F are more clearly detected in Figure 8c than in Figure 8f, although they are closer to the ultrasound excitation position in Figure 8f.

Through Figure 8, when a defect, such as G, is deeper, the ultrasound excitation position should be closer. The vibrothermography results depend on the defect depths, positions and the specimen structures. A modulation ultrasound excitation can generate higher power and might lead to more detection results. An analytical simulation may contribute to the precise study of vibrothermography, through which the more suitable ultrasound excitation position could be determined [22].

## 6. Thermographic Probability of Detection

The reliability of a NDT technique is important to the overall assessment of the inspection. The probability of detection (PoD) curve is the most generally accepted method to value the inspection capability of NDT [23]. However, compared to traditional NDT techniques, such as ultrasonic c-scan, for which has a significant amount of reliable studies have been conducted, the rmographic probability of detection (thermoPoD) was poorly documented in the open literature [19]. Therefore, thermoPoD is meaningful to the thermographic techniques assessments and is being increasingly studied. A PoD curve is a function of a defect characteristic. The characteristic includes the defect size, depth, *etc.*, which are acquired or estimated from inspection data. It is well known that the defect depth and size significantly influence the inspection results. Therefore, a PoD curve is usually studied as a function of aspect ratio r(D/d). The PoD function can be formulated either by the quantitative response data or the hit/miss data (also called binary data). The data can be treated either as a continuous signal response $a^{{}^{\prime }}$, or as a discrete hit/miss response. In the first case, the PoD is obtained from the correlation of variable $a^{{}^{\prime }}vs.\;a$, where *a* denotes defect characterization, such as size, area and aspect ratio. In the second case, data are organized such that a defect is either detected (hit = 1) or not (miss = 0) [10].

In this article, the hit/miss (binary) data based on the comparative study results were chosen to conduct thermoPoD analysis. For example, the inspection result is recorded as either detected (=1) or not (=0) for every inspection and image processing method. Table 4 shows the thermoPoD based on different thermographic techniques and image processing methods.

The study of thermoPoD in this article is not comprehensive; for example, the detection percentage was not given due to the limited quantity of detected defects. Furthermore, further study on thermoPoD by increasing the quantity of defects would be meaningful to provide comprehensive detection capacity. Also further study on statistical thermoPoD, especially continuous thermoPoD, can advance the acceptance of infrared thermography. Finally, the reliability and capability of infrared thermography can be evaluated quantitatively, similar to other NDT approaches. Moreover, it allows for the standardization and characterization of data processing in the industry [19].

## 7. Conclusions

In this article, pulsed thermography, lock-in thermography and vibrothermography were used to detect FODs at different depths in CFRP. In particular, the study on FOD of vibrothermography stimulated at different positions, which was poorly documented in the open literature, was conducted. Then, a comparison was conducted. For pulsed thermography and lock-in thermography, PCT can provide the best inspection results, since PCT is both fast and effective. In contrast, CIS can provide the best results for vibrothermography. Traditional optical excitation thermography cannot detect deep FODs in CFRP. However, vibrothermography performs well. In particular, when a defect is located at a greater depth, the ultrasound excitation position should be closer. The increase of ultrasound excitation power cannot improve the probability of detection, but may damage the specimen. The factors such as the defect depths, positions and the specimen structures affect the detection results together. The complex structure of composites has a significant effect on the detection results. An analytical simulation may contribute to the precise study of vibrothermography, through which the more suitable ultrasound excitation position could be determined.

The thermoPoD study provided a general comparison of the detection capacities. Further study on statistical thermoPoD, especially continuous thermoPoD, is meaningful, which can advance the acceptance of infrared thermography. Finally, the reliability and capability of infrared thermography can be evaluated quantitatively, similar to other NDT approaches. Moreover, it allows for standardization and characterization of data processing used in the industry.

## Figures and Tables

**Table 1:** Theoretical depth.

Lock-in Frequency (Hz)	Theoretical Detective Depth (mm)
3	0.38
1	0.66
0.6	0.86
0.2	1.48
0.1	2.1
0.05	2.96
0.04	3.31
0.03	3.83
0.02	4.69

**Table 2:** The IR camera ’FLIR Phoenix’ technical specifications.

Technical Specification	Explanation/Value
Sensor type	InSb
Waveband	3–5 μm
Pixel resolution	640 × 512
Thermal sensitivity	20 mK

**Table 3:** The ultrasound excitation sepup technical specifications.

Technical Specification	Explanation/Value
Ultrasound frequency	15–25 kHz
Waveform	modulation or pulsed
Minimum modulation frequency	0.1 Hz
Maximum excitation time	10 s
Amplitude	0 to 100%

**Table 4:** Thermographic probability of detection (ThermoPoD).

Thermographic Technique & Image Processing Method	FOD	Delamination
Pulsed thermography & PCT	5/7	1/1
Pulsed thermography & TSR (1st derivative)	4/7	1/1
Pulsed thermography & TSR (2nd derivative)	0/7	1/1
Lock-in thermography & CIS	2/7	1/1
Lock-in thermography & PCT	5/7	1/1
Lock-in thermography & Phase FT	5/7	1/1
Vibrothermography & CIS	7/7	1/1
