# Correction: Liu, B., et al. Quantitative Evaluation of Pulsed Thermography, Lock-In Thermography and Vibrothermography on Foreign Object Defect (FOD) in CFRP. *Sensors* 2016, *16*, doi:10.3390/s16050743

**DOI:** 10.3390/s17010195

**Published:** 2017-01-20

**Authors:** Bin Liu, Hai Zhang, Henrique Fernandes, Xavier Maldague

**Affiliations:** 1 School of Information Science and Engineering, Shenyang University of Technology, 111 Shenliao West Road, Shenyang 110870, China; syuotwenwu@sina.com; 2 Department of Electrical and Computer Engineering, Computer Vision and Systems Laboratory, Laval University, 1065 av. de la Médecine, QC G1V 0A6, Canada; henrique-coelho.fernandes.1@ulaval.ca (H.F.); xavier.maldague@gel.ulaval.ca (X.M.); 3 Department of Mechanical Engineering, Federal University of Uberlandia, 2121 Av. Joao Naves de Avila, Uberlandia 38400-902, Brazil

The authors wish to make the following corrections to this paper [1]:

(1) The title of the article should be changed so that the word ‘Quantitative’ is replaced with ‘Experimental’
(2) Section 3.3 should be removed and replaced with the following:

3.3. Thermographic Signal Reconstruction

The Thermographic Signal Reconstruction (TSR) is widely used in commercial pulsed thermography systems. In TSR, a polynomial function is fitted to each pixel time history to minimize temporal noise. Images created from the instantaneous logarithmic time derivatives of the fit function are typically viewed and analyzed, since the derivative images are much more sensitive to subsurface features than the original data sequence from the IR camera. Individual pixel time history derivatives can be evaluated quantitatively for automated evaluation or measurement of depth or thermal diffusivity. Once the polynomials have been calculated for each pixel, the coefficients may be archived instead of the original data sequence, resulting in a significant degree of data compression.

The implementation of TSR that is used in industrial systems is based on patented and proprietary technology that has optimized specifically for performance with other components in the system. In this study, we have used the basic form of TSR that has been reported in the literature, which we refer to as “B-TSR”. Our polynomial calculation is based on a standard Matlab polynomial fit. Results presented here using B-TSR do not necessarily represent the performance of commercial TSR systems.

(3) The performance of the Thermographic Signal Reconstruction (TSR) method in the experimental study was misrepresented due to the lack of contrast optimization, which was performed on other images in Figure 3. The images 3d and 3e in Figure 3 should be replaced with the contrast optimized results, below: 
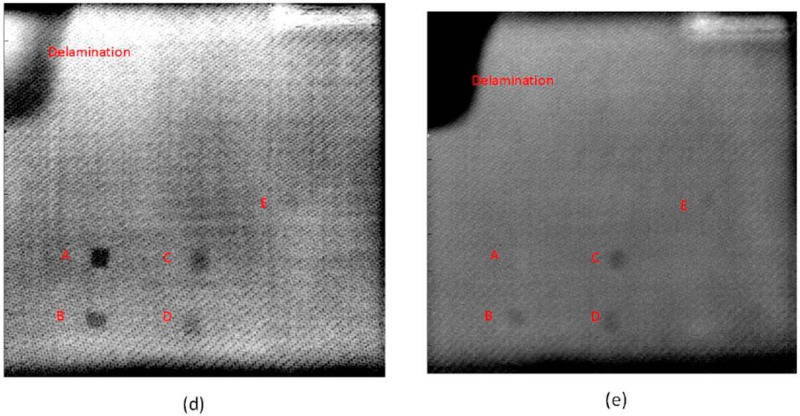

(4) Contrast optimization improves the relative performance of TSR for foreign object defect (FOD) detection, and significantly affects the results of the comparative study. To reflect this, the following sentences on page 5 should be omitted:
  1. But PCT can provide the most inspection details and the best definition
  2. According to the comparison of the images acquired from different image processing methods, PCT is the best solution for this type of defect using pulsed thermography.
(5) The conclusions section should be modified:

The sentence:

“For pulsed thermography and lock-in thermography, PCT can provide the best inspection results, since PCT is both fast and effective”

should be removed, and replaced with

“Pulsed thermography with TSR or PCT processing, or lock-in thermography with PCT, all provided acceptable inspection results”

(6) Table 4 should be removed and replaced with the updated table, below

(7) In Sections 4, 6 and 7, all occurrences of the word ‘TSR’ should be replaced with ‘B-TSR’.
(8) Section 6 (“Thermographic Probability of Detection”) should be removed from the paper;
(9) Table 4 should be moved to the Conclusion section. The caption should read “Performance of Thermographic Methods”
(10) The word “probable” should be eliminated from the following sentence in Section 4.4: The sentence should read as follows:
  “This may indicate the existence of Defect E” (page 5).
(11) The second paragraph in Section 4.4 should be modified to read as follows:

The authors increased the heating times to 120 s for pulsed thermography experiments. However, the longer pulse did not provide better inspection results. This is understandable, since the duration of pulse heating should be set to accommodate the thickness and thermal diffusivity of the sample, and both the 30 and 120 s heating durations were significantly longer than the time required for heat to travel through the sample (~5 s). In particular, TSR is sensitive to transient events while heat is still passing through the sample, therefore the long heating periods and late acquisition used may have cancelled some positive effects of TSR.

The authors apologize for any inconvenience caused to readers. The authors also apologize for the inconvenience caused by our misrepresentation of the TSR method. The manuscript will be updated and the original will remain online on the article webpage.

## Figures and Tables

**Table 4:** Performance of thermographic methods.

Thermographic Technique and Image Processing Method	FOD	Delamination
Pulsed thermography and PCT	5/7	1/1
Pulsed thermography and B-TSR	5/7	1/1
Lock-in thermography and CIS	2/7	1/1
Lock-in thermography and PCT	5/7	1/1
Lock-in thermography and Phase FT	5/7	1/1
Vibrothermography and CIS	7/7	1/1
